# Mapping Vineyard Leaf Area Using Mobile Terrestrial Laser Scanners: Should Rows be Scanned On-the-Go or Discontinuously Sampled?

**DOI:** 10.3390/s16010119

**Published:** 2016-01-19

**Authors:** Ignacio del-Moral-Martínez, Joan R. Rosell-Polo, Joaquim Company, Ricardo Sanz, Alexandre Escolà, Joan Masip, José A. Martínez-Casasnovas, Jaume Arnó

**Affiliations:** 1 Research Group on AgroICT & Precision Agriculture, Department of Agricultural and Forest Engineering, University of Lleida, Rovira Roure 191, Lleida 25198, Spain; jr.rosell@eagrof.udl.cat (J.R.R.-P.); jcompany@alumnes.udl.cat (J.C.); rsanz@eagrof.udl.cat (R.S.); aescola@eagrof.udl.cat (A.E.); jmv@eagrof.udl.cat (J.M.); jarno@eagrof.udl.cat (J.A.); 2 Research Group on AgroICT & Precision Agriculture, Department of Environmental and Soil Sciences, University of Lleida, Rovira Roure 191, Lleida 25198, Spain; j.martinez@macs.udl.cat; 3 Research Group on AgroICT & Precision Agriculture, Agrotecnio Center, Rovira Roure 191, Lleida 25198, Spain

**Keywords:** LAI, LiDAR, mobile terrestrial laser scanner, precision viticulture, vegetation maps

## Abstract

The leaf area index (LAI) is defined as the one-side leaf area per unit ground area, and is probably the most widely used index to characterize grapevine vigor. However, LAI varies spatially within vineyard plots. Mapping and quantifying this variability is very important for improving management decisions and agricultural practices. In this study, a mobile terrestrial laser scanner (MTLS) was used to map the LAI of a vineyard, and then to examine how different scanning methods (on-the-go or discontinuous systematic sampling) may affect the reliability of the resulting raster maps. The use of the MTLS allows calculating the enveloping vegetative area of the canopy, which is the sum of the leaf wall areas for both sides of the row (excluding gaps) and the projected upper area. Obtaining the enveloping areas requires scanning from both sides one meter length section along the row at each systematic sampling point. By converting the enveloping areas into LAI values, a raster map of the latter can be obtained by spatial interpolation (kriging). However, the user can opt for scanning on-the-go in a continuous way and compute 1-m LAI values along the rows, or instead, perform the scanning at discontinuous systematic sampling within the plot. An analysis of correlation between maps indicated that MTLS can be used discontinuously in specific sampling sections separated by up to 15 m along the rows. This capability significantly reduces the amount of data to be acquired at field level, the data storage capacity and the processing power of computers.

## 1. Introduction

Light detection and ranging (LiDAR) is a technology that is becoming widely used by researchers to characterize vineyards and other crops. LiDAR sensors can be airborne or terrestrial and measurements can be performed from stationary or mobile platforms. Different geometric parameters can be measured such as vegetation height, cross-sectional area, canopy volume [1,2,3,4,5,6], and even trunk volume [7]. Other parameters measured with terrestrial laser sensors are the tree area index [5] and the leaf wall area [8]. The accuracy and high density of the generated point clouds make LiDAR information a reliable way to capture detailed geometric characteristics of the canopy structure and analyze plant response to different inputs and conditions [9].

Leaf area and leaf density have been related to multiple agricultural production factors such as solar radiation [10,11], irrigation or water deficit [12,13], fertilization [14], application of plant protection products (PPP) [15,16], and yield and quality of fruits [17,18]. Studies linking LiDAR point clouds or other information provided by remote sensing with the leaf area index (LAI) can be found [5,6,19,20,21], although many of these initial applications were developed for forestry and with airborne LiDAR sensors [22,23].

Among research more specifically about precision viticulture, the spatiotemporal variability of vineyards has been widely studied [24], often using remote sensing techniques [25,26] to map vineyard leaf area [27,28]. However, very little information has been published [29] regarding the mapping of vineyard canopies using ground-based LiDAR sensors, and more importantly, the difficulty of the use and interpretation of the proposed maps makes managing the spatial variability in these cases a remaining challenge.

The main objective of this study was to develop a methodology that allows the use of mobile terrestrial laser scanners (MTLS) in viticulture to estimate and map the LAI. The laser scanner used in this work has been previously designed and validated for use in tree crops [8]. There are two issues covered in this study. The first is the method adopted to process the data. The second is to optimize the use of MTLS in field conditions by replacing the on-the-go scanning along the rows by discontinuous, and more affordable, scanning at only certain sampling points.

## 2. Material and Methods

### 2.1. Field Trial

**Figure 1:**
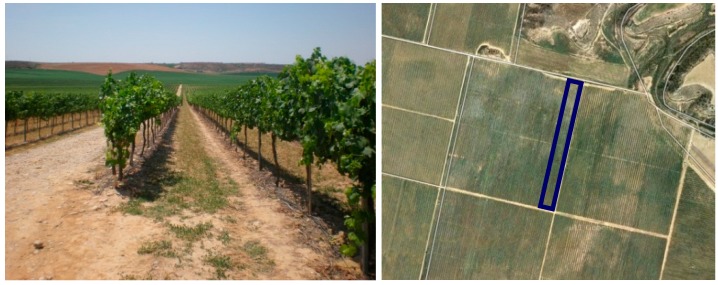
Plot of *Vitis vinifera* L. cv. Syrah (**left**), and location of the six rows scanned with the terrestrial laser scanner (**right**) [32]. The length of the vine rows is 360 m.

A vineyard plot with contrasting vigor was chosen for the field test (Figure 1). The vineyard was located in Raimat (Catalonia, Spain), and was planted in 2002 with the cultivar *Vitis vinifera* L. cv. Syrah. The six rows of the plot where the trial was conducted were oriented north-south, covering a total length of 360 m (approximately 0.70 ha). The vines were trained in vertical shoot position, and were watered by partial rootzone drying. The test was performed at growth stage 79 (majority of berries touching) according to the Biologische Bundesanstalt, Bundessortenamt und CHemische Industrie (BBCH)-Scale [30]. The vines were less vigorous at the northern, highest part of the plot, and more vigorous at the southern area at lower elevation. Given the highly structured spatial distribution of vigor, the plot was very suitable to assess MTLS for detecting the variability of the vineyard. Soils in these fields were classified as Typic Haploxerepts and Fluventic Haploxerepts [31]. The Typic Haploxerepts, located in the highest part of the plot, presented a paralithic contact within the first 50 cm, which could be the main reason for the differences in vigor detected in the plot.

### 2.2. Mobile Terrestrial Laser Scanner

The sensor used in this research was described in detail in [8]. In fact, this work only involves the field application of the abovementioned system, comprising the LMS-200 (SICK AG, Waldkirch, Germany) to acquire data on vegetation, a real-time kinematic global positioning system (RTK-GPS) to georeference these data, and an inertial measurement unit (IMU) to correct deviations due to movements of the sensor. The latter was an MTi sensor (XSens, Ans Enschede, The Netherlands) and provided current roll and pitch rotation based on the plumb line and the earth’s magnetic field. Position and yaw of the MTLS were estimated using RTK-GPS information provided by a GNSS 1200 (Leica Geosystems AG, St. Gallen, Switzerland). All three devices supplied information at different output rates. In our case, RTK-GPS worked at 5 Hz, MTLS worked at 15 Hz, and the IMU worked at 100 Hz. All information was timestamped so data could be synchronized, and then it was transferred to an on-board central computer via the RS-232 protocol. A MATLAB-based program was used to control the sensors and acquire data.

**Figure 2:**
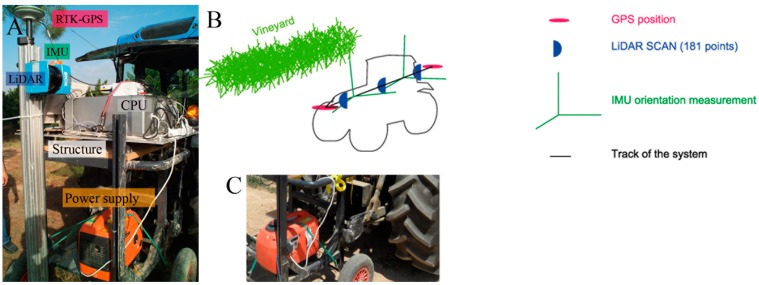
Image of the different sensors (**A**); interaction of the MTLS components (**B**); and connection to the three-point hitch of the tractor (**C**).

All the MTLS components together with a power supply unit were mounted in a structure connected to the three-point hitch of a tractor (Figure 2). The choice of using a tractor allows to better drive around the vineyard and to easily choose constant travel speed. In the present work, the travel speed was set in about 3.6 km·h^−1^.

**Figure 3:**
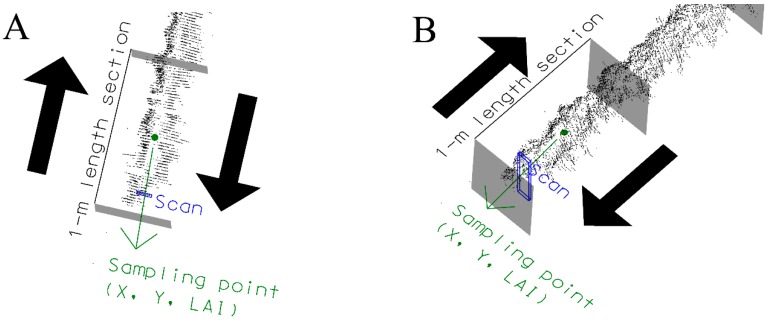
1-m long section of the row including *scans* obtained from both sides of the row. (**A**) and (**B**) are views of the same scene from different perspective. The LAI estimation is assigned to the *sampling point*.

A basic feature of the MTLS used was a LiDAR sensor emitting a laser beam used to measure the distance between the objects and the sensor, based on the time-of-flight principle [33]. This time of flight is the travel time of the laser beam to go from and return to the LiDAR sensor after being reflected by the object of interest. Specifically, a two-dimensional fan-shaped scan was obtained (sweep angle of 180°) since the laser beam was pulsed with an angular resolution of 1°. In this way, the scanner provided up to 181 distances (points of interception) in each scan. When scanning a row, the scanner field of view was oriented towards the right hand side according to the forward direction, and only one side of the row could be scanned at a time (Figure 2). Thus, each vegetation row needed to be scanned from both sides. In a subsequent step, the sensor data was corrected with the IMU data and UTM coordinates were assigned to each interception point according to the methodology used in [8]. Once the point cloud was created, 1-m long sections were delimited including points obtained from both sides of the row (Figure 3). Then, a LAI estimation algorithm was applied as described in the next section.

### 2.3. LAI Estimation

**Figure 4:**
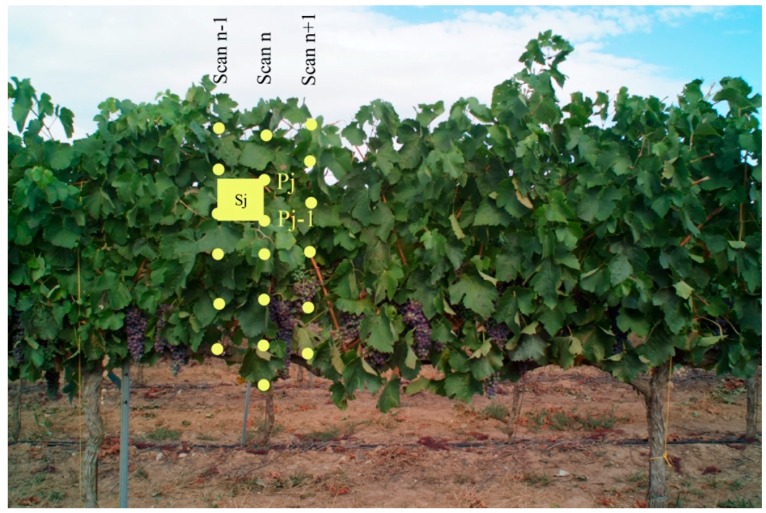
Intercepted points generated by consecutive scans and specific pixelated area (Sj) assigned to one of interception points (Pj).

A pixelated area (S_j_) was assigned to each of the interception points (Figure 4), calculated as the product of the vertical distance to the previous point and the horizontal distance to the previous scan [8]. The sum of the pixelated areas in each scan was finally assigned to a representative point whose coordinates were the average of the coordinates of the interception points. This procedure was repeated for each of the scans included in a 1-m length section (Figure 3).

**Figure 5:**
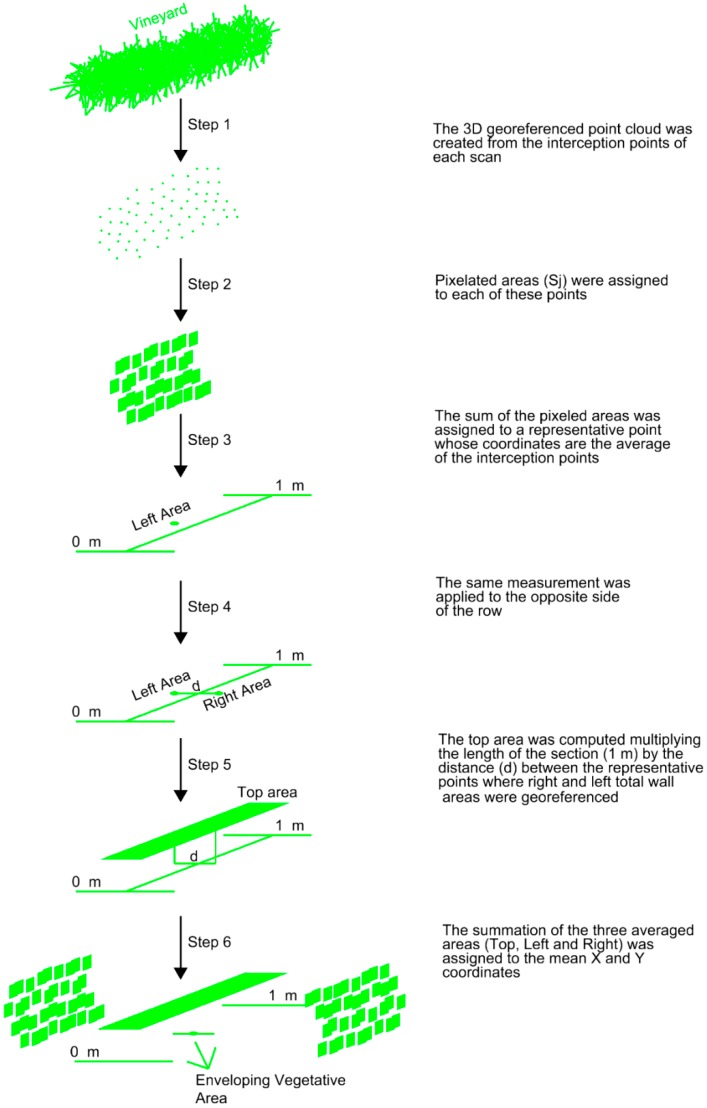
Diagram of data processing to compute the envelope vegetative area of the 1-m length section.

As a result, the total leaf wall area from one side of the row was computed as the sum of the pixelated areas of each scan and georeferenced at the average coordinates of the scans in 1-m length section. The same was repeated for the opposite side of the row. The ultimate goal was to obtain the so-called enveloping vegetative area of the canopy, consisting of the sum of the two leaf wall areas (excluding gaps) and the area that encloses the top of the row. The top area was computed considering the distance between the points where the right and left leaf wall areas were georeferenced. The training system of the plot allows for an important canopy development in width. This parameter should be taken into account since it represents a large leaf area. The inclusion of row width data is the main difference to previous work in this respect [8]. Figure 5 shows the different steps of the process. LAI was then calculated using the following expression:

(1) $$LAI=\frac{(S_{L}+S_{R}+S_{T})\cdot C}{d_{r}\times L}$$

where *S_L_* (m^2^) and *S_R_* (m^2^) were the left and right leaf wall areas, respectively, *S_T_* (m^2^) was the top area, *C* (dimensionless) was the ratio of the foliar surface to the enveloping area, which is equal to 1.70 in the vineyard (“unpublished report”), *d_r_* was the row spacing (m), and *L* (m) was the section length (1 m in the present case). Since the areas were calculated for 1-m length sections, the final result was the corresponding LAI (m^2^·m^−2^). The georeferenced LAI of this 1-m length section is called *sampling point* in the current research work (Figure 3). This process allowed georeferenced LAI values to be generated according to 1 m equidistant points placed along the line of grapevine trunks.

Clearly, point clouds contained points on leaves, trunks and branches. However, no classification is performed because the point clouds only had position information (no intensity or color is provided by the MTLS system). Nevertheless, the *C* parameter used in Equation (1) has been obtained taking into account the effect of trunk and branches.

**Figure 6:**
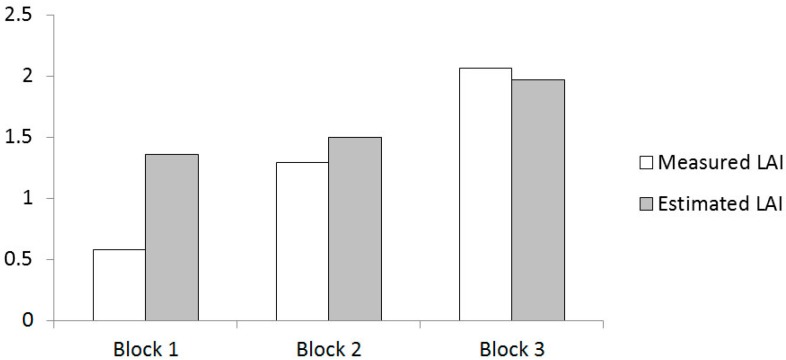
Measured and estimated LAI in three different contrasting vineyard blocks.

To validate the method for estimating the LAI, three vineyard blocks of different contrasting vigor were selected within the field. Each block covered a row length of 2 m (distance between two consecutive trunks), and was scanned on both sides with the MTLS. After scanning, the three blocks were manually defoliated to measure the actual values of LAI to compare with the values provided by the electronic measurement. Results are shown in Figure 6.

The MTLS performed good estimates of the LAI for intermediate and high vigor vines, with an error between 4% and 16%. However, in vines with reduced vigor, the error was high, probably due to the horizontal scanning resolution used [33] (a scan every 7 cm along the row). This distance between scans may have been too long in vines with poor leaf development, computing as effective leaf wall area zones in the canopy with a considerable percentage of gaps. This should be improved in future versions of the MTLS.

**Figure 7:**
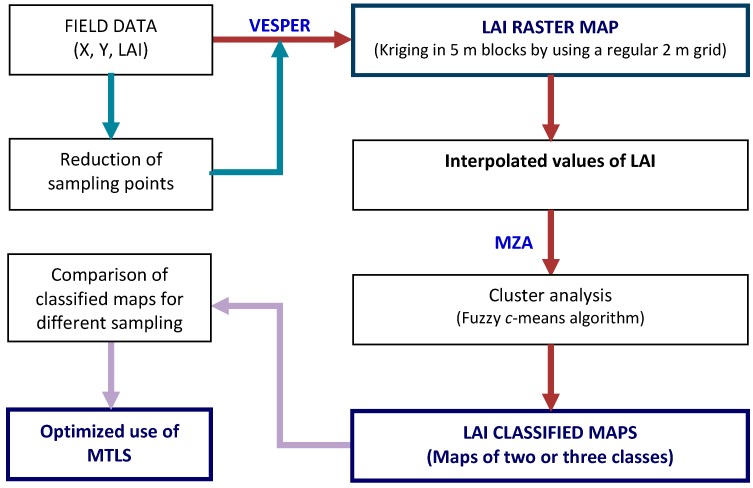
Flowchart for obtaining and comparing LAI raster maps.

Finally, a LAI raster map was created using the previously validated data and applying a geostatistical method of spatial interpolation (Figure 7). VESPER software [34] was used for ordinary kriging and ArcGIS 10.1 (ESRI, Redlands, CA, USA) to import and map the interpolated values. Specifically, a kriging on a 5 m block basis was used having previously adjusted the global variogram model that best fitted the data (spherical model).

Next, an unsupervised classification algorithm (fuzzy *c*-means) was applied to the interpolated data in order to cluster the LAI values and then generate classified LAI maps. Management Zone Analyst (MZA) [35] software was used for this purpose. According to previous research on grape quality for selective harvesting purposes [36], the winery decided to work with only two vigor classes to produce two different wine qualities. Additionally, they also apply plant protection products variably based on the use of two or three different doses as much. They still use conventional sprayers, and more than two or three doses (classes) within the same plot would be difficult to handle. Hence, we opted for LAI classification in two or three classes, although the sensor allows working with higher resolution to obtain a larger number of classes.

## 3. Results and Discussion

**Figure 8:**
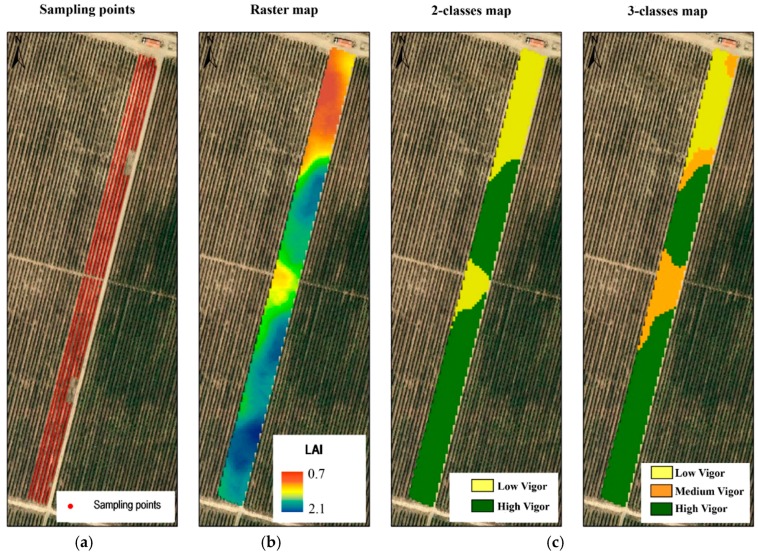
(**a**) Sampling points, (**b**) LAI raster (interpolated) map and (**c**) LAI classified maps (2 classes and 3 classes) in an area of 0.70 ha within a plot in a vineyard.

Figure 8 shows the LAI raster map. As expected, the spatial variability was remarkable (coefficient of variation of 24%), but more interesting was that the variation in LAI was highly structured, allowing the delimitation of well-defined and compact areas within the field. Adopting the approach suggested by [35], two additional maps were obtained based on the classification of LAI into two and three classes of vigor (Figure 8). 

From the perspective of site-specific vineyard management, LAI classified maps provide a clear view of areas with different canopy development and, consequently, areas that could potentially be managed differently. To mention one example, LAI maps can be used in the variable-rate application of pesticides to adjust the dosage to the canopy characteristics.

However, it is difficult to extensively use this technology in commercial fields. The time required to measure a whole plot and the large amount of data generated are the two major drawbacks of using MTLS in agriculture on a commercial basis, especially when continuously scanning the rows of the plot. Therefore, an alternative to the use of on-the-go MTLS is to discontinuously scan the canopy according to a specific sampling strategy along the vine rows. The results of this study are shown in the following section.

### Sampling Optimization of MTLS Measurements

When the MTLS was used on-the-go, that is, scanning continuously along the rows, a total number of 2146 LAI sampling points were obtained (Figure 8). This method took approximately 2 h and generated 1 GB of information per hectare, considering that the scanned length at each sampling point was 1 m.

**Figure 9:**
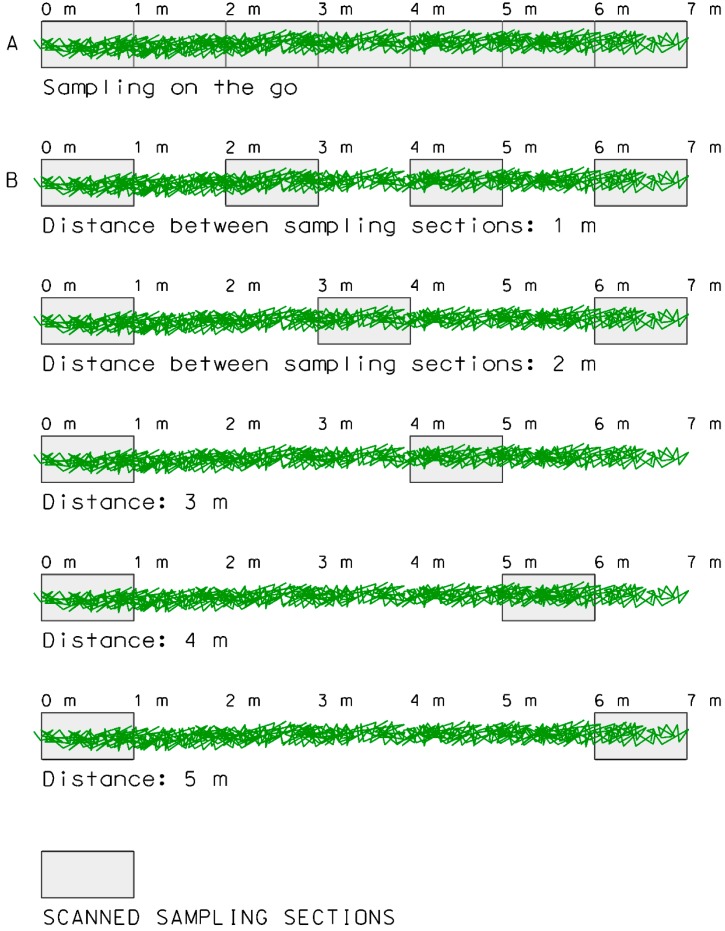
Different modes of operation of a MTLS along a row of vines. The rectangular area indicates the scanned sampling sections. (**A**) On-the-go scanning mode; (**B**) discontinuous scanning mode separating the sampling sections 1 m.

To reduce the acquired information, the discontinuous operation of MTLS was simulated. In this mode, the scanner would only be used every few meters, depending on the distance between sampling points. A graphical scheme of different operating modes is shown in Figure 9. 

When the vineyard was scanned on-the-go (Figure 9A), the sampling points were located one after the other. The acquired data could be reduced by 50% by separating the sampling sections by a distance equal to the scanned length (Figure 9B). Further reductions in input information would be achieved by increasing the distance between sampling sections. However, there is probably a limit to this procedure, and the question is how much this distance can be increased without affecting the quality of the generated maps. Starting with the original data (Figure 9), sampling sections and the corresponding sampling points were progressively removed, simulating the intermittent operation of the MTLS. A total of 10 different patterns were simulated depending on the separation distance: 1, 2, 3, 4, 5, 10, 15, 20, 25 and 30 m. Some of these distances are shown in Figure 9. For each of these sampling point sets, a LAI raster map was obtained following the methodology described in Figure 7. Then, the resulting maps were classified into two or three vigor classes. The final step was to assess the correlation between the original classified maps (on-the-go sampling) and the classified maps resulting from the use of fewer data (discontinuous sampling along the rows).

**Figure 10:**
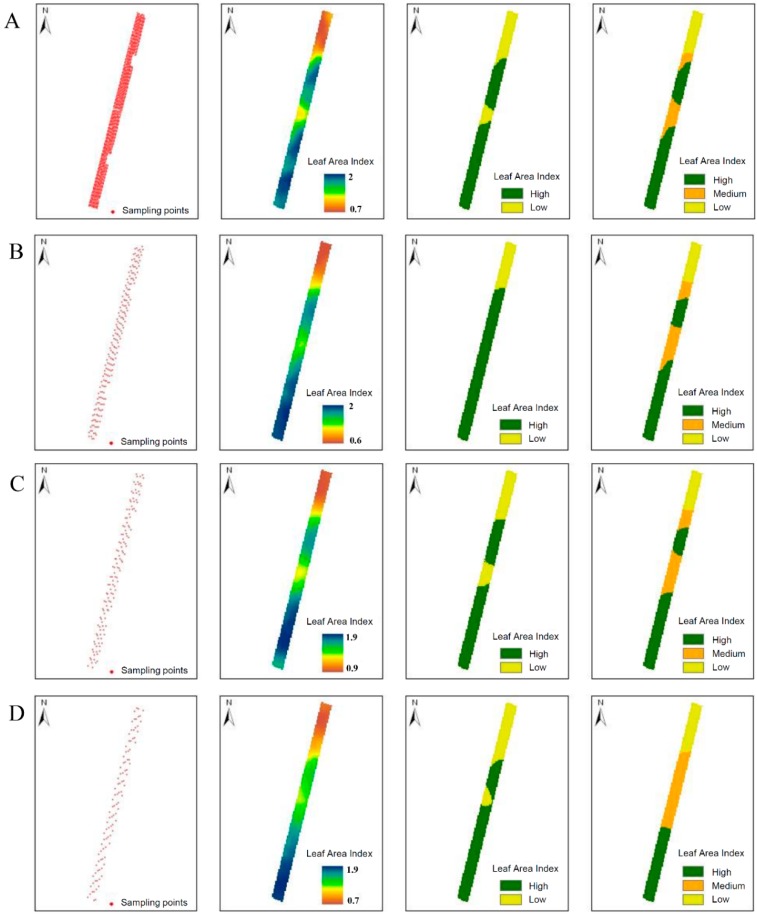
Sampling points, LAI raster maps, and LAI classified maps for different MTLS sampling schemes along the rows. The sampling sections are separated 1 m (**A**); 10 m (**B**); 15 m (**C**); and 20 m (**D**).

Figure 10 shows the maps obtained after reducing the number of sampling sections. In particular, the sampling schemes corresponded to distances of 1, 10, 15, and 20 m between scanned sections. The LAI raster maps were quite similar to the original map (on-the-go sampling). However, differences were more evident when comparing the LAI classified maps. Visual analysis of Figure 10 shows that the discrepancy between classified maps obtained by discontinuous sampling and the equivalent maps obtained with continuous sampling (Figure 8) increased with decreasing sampling points, that is, when scanned vines were separated by a greater distance. This result is expected and to some extent justifiable, but it is more important to quantify the degree of discordance as the scanned data are omitted. Comparing the initial LAI classified maps (Figure 8) with the corresponding maps obtained with a discontinuous sampling (Figure 10) was performed using the Kappa coefficient (*k*):

(2) $$k=\frac{P_{o}-P_{e}}{1-P_{e}}$$

where *P_o_* is the observed class concordance between pixels from different maps, and *P_e_* is the expected agreement.

Table 1 shows the results from the comparison between maps (analysis of the degree of concordance). The number of sampling points was drastically reduced when increasing distance between sampling sections. However, good agreement with the original classified maps was achieved when the distance between sampling sections did not exceed 15 m. Both LAI classified maps (two and three classes) had a Kappa coefficient above or close to 0.8. The best results were produced with higher sampling point densities (distances between sampling sections shorter than 5 m), but probably the best option is to keep a distance between 10 and 15 m not to saturate the data acquisition system. This implies obtaining 278 and 192 sampling points per hectare, respectively, so reliable raster maps could then be obtained by ordinary kriging. In contrast, separating sampling sections by a greater distance (20 m or more) is not recommended when using MTLS.

**Table 1:** Analysis of the correlation between the LAI classified maps obtained by on-the-go scanning (with a total of 2146 sampling points, N) and those obtained discontinuously scanning according to various sampling schemes.

Distance between Sampling Sections (m)	Number of Sampling Points	Kappa Coefficient 2-Class Vigor Maps	Kappa Coefficient 3-Class Vigor Maps
1	N/2 = 1073	0.95	0.91
2	N/3 = 715	0.95	0.88
3	N/4 = 537	0.75	0.89
4	N/5 = 429	0.93	0.70
5	N/6 = 358	0.82	0.85
10	N/11 = 195	0.73	0.80
15	N/16 = 135	0.90	0.75
20	N/21 = 103	0.75	0.56
25	N/26 = 83	0.75	0.50
30	N/31 = 70	0.54	0.59

Another case of use could be the study over the time of the vineyard vigor (multiple-scans scenario). A first complete measurement could be done at the beginning of the season. After that, and once established the distance sampling better adapted to the data, the optimal sampling could be applied in following measurements. Indeed, in some cases it may be advisable to vary the sampling strategy for different areas within the plot.

## 4. Conclusions

MTLS can be used in viticulture to obtain LAI maps. However, on-the-go sampling generates a large amount of data that can be difficult to process and store. One solution to this problem is the discontinuous use of MTLS based on scanning specific sampling sections. Each section should be 1 m long, and the distance between sampling sections should not exceed 15 m to obtain a reliable LAI map. A greater distance yields maps that do not fit the actual spatial variability within the plot and, consequently, are not suitable for precision viticulture practices. More reliable maps can be obtained when MTLS are used more intensively, separating the sampling sections by no more than 5 m, but at the cost of acquiring, storing and processing large amounts of data. In any case, the remaining issue is to avoid the overestimation of LAI in areas where vines have low vigor. This requires reducing the distance between scans in order to better account for canopy gaps.
